# Suppression in PHLPP2 induction by morin promotes Nrf2-regulated cellular defenses against oxidative injury to primary rat hepatocytes

**DOI:** 10.1016/j.redox.2015.10.002

**Published:** 2015-10-19

**Authors:** Fatima Rizvi, Alpana Mathur, Shagun Krishna, Mohammad Imran Siddiqi, Poonam Kakkar

**Affiliations:** a Herbal Research Laboratory, Food Drug and Chemical Toxicology Group, Council of Scientific and Industrial Research-Indian Institute of Toxicology Research (CSIR-IITR), Post Box No. 80, M.G. Marg, Lucknow 226001, Uttar Pradesh, India; b Molecular and Structural Biology Division, Council of Scientific and Industrial Research-Central Drug Research Institute (CSIR-CDRI), Lucknow 226031, Uttar Pradesh, India; c Academy of Scientific and Innovative Research, CSIR-IITR campus, Lucknow, India

**Keywords:** AIF, apoptosis inducing factor, APAP, acetaminophen, ARE, antioxidant redox element, DCFH-DA, 2′,7′-dichlorofluorescein diacetate, DHE, dihydroethidium, ELISA, enzyme-linked immunosorbent assay, FITC, fluorescein isothiocyanate, GAPDH, glyceraldehyde phosphate dehydrogenase, GPx, glutathione peroxidase, GR, glutathione reductase, GSH, glutathione, GSSG, glutathione disulfide, GST, glutathione S-transferase, HO1, heme oxygenase 1, JC-1, 5,5′,6,6′-tetrachloro-1,1′,3,3′-tetraethylbenzimidazolyl carbocyanine iodide, Keap1, Kelch-like ECH-associated protein-1, MTT, 3-(4,5-dimethylthiazol-2-yl)-2,5-diphenyltetrazolium bromide, NQO1, NAD(P)H quinine oxidoreductase 1, Nrf2, Nuclear factor erythroid 2-related factor 2, PHLPP2, PH-domain and Leucine rich repeat protein phosphatase 2, PVDF, polyvinylidene fluoride, ROS, reactive oxygen species, SEM, standard error of the mean, siRNA, small interfering RNA, tBHP, tert-butyl hydroperoxide, TrxRed, thioredoxin reductase, Oxidative stress, PHLPP2, Fyn kinase, Nrf2, Flavonoid

## Abstract

Recent advances indicate a possible role of phytochemicals as modulatory factors in signaling pathways. We have previously demonstrated PHLPP2-mediated suppression of Nrf2 responses during oxidant attack. The present study was designed to explore Nrf2-potentiating mechanism of morin, a flavonol, via its possible role in intervening PHLPP2-regulated Akt/GSK3β/Fyn kinase axis. Efficacy of morin was evaluated against oxidative stress-mediated damage to primary hepatocytes by tert-butyl hydroperoxide (tBHP) and acetaminophen. The anti-cytotoxic effects of morin were found to be a consequence of fortification of Nrf2-regulated antioxidant defenses since morin failed to sustain activities of redox enzyme in Nrf2 silenced hepatocytes. Morin promoted Nrf2 stability and its nuclear retention by possibly modulating PHLPP2 activity which subdues cellular Nrf2 responses by activating Fyn kinase. Pull-down assay using morin-conjugated beads indicated the binding affinity of morin towards PHLPP2. Molecular docking also revealed the propensity of morin to occupy the active site of PHLPP2 enzyme. Thus, dietary phytochemical morin was observed to counteract oxidant-induced hepatocellular damage by promoting Nrf2-regulated transcriptional induction. The findings support the novel role of morin in potentiating Nrf2 responses by limiting PHLPP2 and hence Fyn kinase activation. Therefore, morin may be exploited in developing novel therapeutic strategy aimed at enhancing Nrf2 responses.

## Highlights

• Cytoprotection against tBHP-evoked oxidative stress by morin is Nrf2-regulated.
• Morin prevents Nrf2-destabilization via PHLPP2-Akt-GSK3β-Fyn kinase pathway.
• In silico docking studies confirmed PHLPP2 activity inhibition by morin.
• Morin mitigates APAP induced cytotoxicity by suppressing PHLPP2 pathway.

## Graphical abstract

**Figure:**
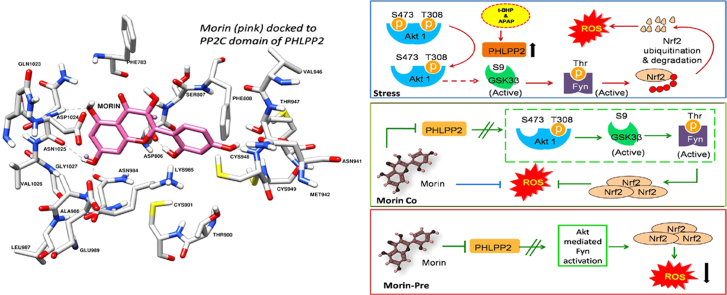

## 1 Introduction

Reactive oxygen and nitrogen species (ROS/RNS) have the capacity to act as signaling moieties involved in maintenance of cellular homeostasis [1]. However, their accumulation within the cell may lead to oxidative imbalances, altered homeostasis and subsequent cell death. Such an occurrence is prevented through a coordinated defense network, such as the Antioxidant Response Element (ARE)/Nrf2 pathway, that guards the cellular makeup from a possible oxidative insult. When confronted with oxidant challenge, Nuclear factor-erythroid 2 p45-related factor 2 (Nrf2), translocates to the nucleus, where, in association with other proteins, it keeps a panel of cytoprotective genes under its tight regulation [2]. Nrf2 is activated and translocated to the nucleus even when a mild rise in oxidative load is sensed [3]. However, any irregularity in its mechanism potentiates free radical-induced stress, thereby disturbing normal cellular physiology.

Perturbed Nrf2 activity has been associated with the progression of a number of pathological conditions involving oxidative imbalances [4], [5], [6]. Our previous findings on oxidative toxicity imposed by *tert*-butyl hydroperoxide (tBHP) [7] documented suppression in cellular Nrf2 responses due to induction of PH domain Leucine-rich repeat Protein Phosphatase 2 (PHLPP2) which ultimately led to GSK3β and Fyn kinase activation. PHLPP isozymes (PHLPP1 and PHLPP2) regulate the PI3K signaling by selective dephosphorylation (of Akt Ser473 residue) and hence down-modulation of Akt activity [8]. The phosphatidylinositol-3 kinase (PI3K) signaling is well known to play a pivotal role in maintaining cellular homeostasis. Due to its central role in deactivation of Akt, PHLPP is viewed as an attractive drug target for positive or negative regulation of PI3K signaling in disease [9]. Owing to the inhibitory effect of PHLPP2 on Nrf2 activity [7], checking PHLPP2 activity may prove beneficial under circumstances where Nrf2 signaling is muted due to increased Nrf2 destabilization.

The functional integrity of Nrf2-regulated antioxidant and detoxification system is of utmost importance for the maintenance of hepatic physiology. At present, dietary phytoconstituents that could assist in alleviating oxidative stress-associated repercussions are increasingly being sought-after [10], [11]. Plant-derived phytochemicals are now widely accepted as important dietary factors that boost our health by protecting cells against oxidative damage. The wide range of health promoting effects of these phytochemicals has often been attributed to the induction of Nrf2 pathway [12]. Morin (2′,3,4′,5,7-pentahydroxyflavone), a flavonol, has been shown to possess a wide array of biological activities including anti-oxidant [13], anti-hyperglycaemic [14] and hepato-protective [15] properties. Though its role as an antioxidant or free-radical scavenger in promoting cytoprotection has been established, no study has yet addressed its function as modulator of Nrf2-mediated signaling pathways to counter cytotoxicity arising due to oxidative stress.

Hence, the present study was aimed at defining the mechanistic involvement of morin in mitigation of dysregulated Nrf2 responses during an event of oxidant attack by employing tBHP as an oxidative stress generating agent. Further, the cytoprotective mechanism of morin investigated in oxidatively compromised (tBHP-treated) hepatocytes was also confirmed in hepatocytes exposed to cytotoxic concentrations of acetaminophen (APAP).

## 2 Materials and methods

### 2.1 Materials and reagents

Antibodies and chemicals including ERK1/2 (4695), phospho-ERK1/2 (9101), SAPK/JNK (9252), phospho-SAPK/JNK (4668), phospho-Akt Ser473 (4060), phospho-Akt Thr308 (2965), phospho-GSK3β Ser9 (9323), phospho-PDK1 Ser241 (3438), phospho-PTEN Ser380 (9551), Cox-IV (11967), anti-rabbit Alexafluor 555 conjugate (4413) were purchased from Cell Signaling Technology (Danvers, MA). Antibodies against caspase-12 (ab62484), HMGB1 (ab79823) and NALP3 (ab17267) were obtained from Abcam (Cambridge, UK). FBS, 100X antimycotic and antibiotic solution, Collagenase (type IV), OPTI-MEM reduced serum medium, William's medium E, Lipofectamine RNAiMAX Transfection Reagent, CellTracker™ Green CMFDA dye and antibody against caspase-1 (AHZ0082) were procured from Invitrogen (Carlsbad, CA). Silencer Select Pre-designed siRNA against Nfe2l2 (ID:s136127), negative control unlabeled siRNA were supplied by Ambion (Austin, TX). Chemicals and antibodies like Nrf2 (sc-30915), Keap1 (sc-33569), β-actin (sc-81178), GAPDH(sc-25778), Ubiquitin (sc-166553), Lamin b (sc-6216), HO1 (sc-10789), NQO1 (sc-16463), Akt1 (sc-5298), Fyn kinase (sc-16-G), caspase-3 (sc-7148), caspase-9 (sc-8355), Bax (sc-493), Bcl2 (sc-492-G), cytochrome c (sc-8385), agarose conjugated phospho-Thr antibody (sc-5267AC), secondary antibodies, normal goat sera and Protein A/G PLUS Sepharose beads were purchased from Santa Cruz Biotechnology (Santa Cruz, CA). CNBr-activated Sepharose beads were purchased from GE-Healthcare (GE Healthcare BioSciences Ltd., Kowloon, Hongkong). All other reagents or chemicals including acetaminophen, morin hydrate, tBHP, PHLPP2 antibody (SAB1300919), anti-goat FITC conjugated secondary antibody (F7367), Hoechst 33258 and dexamethasone were procured from Sigma (St Louis, MO, USA) unless otherwise mentioned.

### 2.2 Primary rat hepatocytes isolation, culture and treatment

Primary hepatocytes were isolated from male wistar rats weighing 100–120 g, 6- to 8-week-old through portal vein collagenase perfusion of liver as per the method of Seglen [16]. Rats were procured from the animal house of CSIR-Indian Institute of Toxicology Research. All rats were housed in environmentally controlled rooms under standard conditions of humidity 60–70%, temperature 25±2 °C and a 12 h light/dark cycle. Animal handling in all experimental procedures was approved by the Institutional Animal Ethics Committee (Ref no. ITRC/IAEC/20/09-01/11-33/12). Hepatocytes were seeded on collagen-coated surface and were cultured for 4 h in William's medium E supplemented with 50 nmol/l dexamethasone and 5% fetal bovine serum (FBS) in addition to 2 mmol/l glutamine. Thereafter, the cells were cultured in the same medium lacking dexamethasone and FBS. tBHP was freshly prepared in culture medium while morin stock solution was prepared in dimethyl sulfoxide (DMSO) and thereafter diluted in culture medium taking care that DMSO concentration does not exceed 0.01% in any of the treatments. Cytoprotective efficacy of morin against tBHP was tested by treating hepatocytes with different concentrations of morin in combination with tBHP stress. In case of morin pre-treatment, hepatocytes were exposed to selected concentration of 10 µM morin for different time periods and washed with fresh media before subsequent treatment with tBHP. In experiments involving acetaminophen (APAP), hepatocytes were treated with 1 mM APAP (prepared by dissolving in media) for 90 min. A concentration of 10 µM morin was tested for its efficacy against APAP. Cell viability was estimated by determining the reduction of 3-(4,5-dimethylthiazol-2-yl)-2, 5-diphenyltetrazolium bromide (MTT) to formazan. The absorbance corresponding to that of untreated control cells was assumed as 100% cell viability.

### 2.3 Measurement of intracellular reactive oxygen species (ROS)

Intracellular ROS generation was estimated using 2′,7′-dichlorofluorescein diacetate (DCFH-DA). Briefly, hepatocytes plated at a density of 10,000 cells/well were incubated with DCFH-DA (10 µM) for 30 min at 37 °C prior to treatment with tBHP and/or morin. In case of morin pre-treatment, DCFH-DA (10 µM) was added 30 min prior to end of 3 h incubation with morin. Measurements were taken at different time periods during the course of the treatment schedule using Varioskan Flash Multimode microplate reader (Thermo Fisher Scientific) at 485 nm excitation and 530 nm emission.

### 2.4 Measurement of glutathione (GSH) levels

For glutathione estimation, CellTracker™ Green CMFDA dye (5-Chloromethylfluorescein Diacetate; Invitrogen) was used, which detects GSH with a specificity of 95%. Cells were incubated with 5 µM CMFDA at 37 °C prior to treatment and measurements were made at different time periods at excitation wavelength of 492 nm and emission wavelength of 517 nm using Varioskan Flash Multimode microplate reader (Thermo Fisher Scientific).

### 2.5 Evaluation of mitochondrial membrane potential

Cells were treated with 5 µM 5,5′,6,6′-tetrachloro-1,1′,3,3′-tetraethylbenzimidazolyl carbocyanine iodide (JC-1) in a manner similar to that mentioned above. The green to red shift in fluorescence was measured at different time periods during the course of the treatment using Varioskan Flash Multimode microplate reader. The results are expressed as red/green fluorescence ratio measured at 515 nm/529 nm (excitation/emission) for green and 515 nm/530 nm (excitation/emission) for red JC-1 aggregates.

### 2.6 Measurement of intracellular calcium levels

Fura 2-AM dye was used to evaluate intracellular calcium levels. The hepatocytes were treated with 5 µM Fura 2-AM for 30 min at 37 °C and treated in a similar manner as for ROS estimation. The calcium-free or unbound fura dye was monitored at 380 nm excitation and 510 nm emission wavelengths while the calcium-bound dye was measured at 340 nm excitation and 510 nm emission wavelengths using Varioskan Flash Multimode microplate reader. The 340/380 ratio was calculated to determine calcium levels at different time intervals during treatment period.

### 2.7 DCF/DHE staining

For fluorescent microscopic detection of ROS, hepatocytes were stained with 10 µM DCFH-DA and 5 µM DHE. Hoechst 33258 was used to stain nuclei and observed under Nikon ECLIPSE 80*i* upright microscope (Nikon Corporation, Japan) using 10× objective magnification.

### 2.8 Measurement of caspases activity

The activity of caspases namely, caspase-3, caspase-9 and caspase-12 were determined using the Caspase-3 Colorimetric Assay Kit, Caspase-9 Colorimetric Assay Kit and Caspase-12 Fluorometric Assay Kit (BioVision, Inc., Mountain View, CA), respectively, as per the manufacturer's instruction.

### 2.9 Immunocytochemistry

Cells were washed with cold 0.01 M PBS (pH 7.2) and fixed in 4% paraformaldehyde for 10 min. The cells were then washed with 0.05% glycine in PBS and then permeabilized with 1% Triton X-100 (v/v in PBS) for 15 min followed by overnight incubation with anti-Nrf2 antibody at a dilution of 1:200 in PBS, the cells were rinsed thrice with PBS for 5 min each. This was followed by 3 h incubation in FITC-conjugated secondary antibody at 1:500 dilution. Nuclei were counter-stained with Hoechst 33258 (1 µM) for 15 min. Cells were observed under Nikon ECLIPSE T*i-S* inverted microscope (Nikon Corporation, Japan) under 40× objective.

### 2.10 Sub-cellular fractionation

Isolation of nuclear and cytoplasmic fractions from hepatocytes was achieved using NE-PER extraction kit (Pierce, Thermo Scientific). Concentration of protein was determined using Bicinchoninic acid (BCA) method.

### 2.11 Enzyme activities

To obtain cell lysate for estimation of enzyme activity, primary hepatocytes were suspended in 50 mM Tris–Cl (pH 7.4) containing 0.1% Nonidet P-40, 150 mM NaCl and Protease inhibitor cocktail. The cells were gently agitated at 4 °C for about 1 h and thereafter centrifuged at 16,000*g* to obtain cell lysate

*Glutathione reductase activity (EC 1.8.1.7)*: Glutathione reductase activity was measured as the amount of NADPH consumed in reducing GSSG to GSH. Briefly, the activity was measured spectrophotometrically (Ultrospec 3100pro) at 340 nm after adding 1 mM GSSG to a reaction system containing 0.2 mM NADPH in 0.1 M Tris–Cl (pH 7.5) and cell lysate containing 40 µg protein sample. Absorbance was recorded at every 30 s interval for 3 min at 25 °C (Extinction coefficient for NADP^+^=6.22 mM^−1^ cm^−1^).

*Thioredoxin reductase activity (EC 1.8.1.9)*: Thioredoxin reductase activity was determined spectrophotometrically using 5,5′-dithiobis (2-nitrobenzoic acid) (DTNB). Thioredoxin reductase catalyses the reduction of oxidized thioredoxin while converting DTNB to TNB (5-Thio-2-Nitrobenzoic Acid) in the process. Reaction mixture containing 0.25 mM NADPH and 7 mM EDTA in 0.2 M sodium phosphate buffer (pH 7.2) was prepared. Protein sample (40 µg) was added to 400 µl reaction mixture and the reaction was initiated by adding 400 µl of 8 mM DTNB in a cuvette. Increase in absorbance was measured on Ultrospec 3100pro UV/visible spectrophotometer (Amersham Biosciences, Sweden) at 412 nm at a regular interval of 60 s for about 5 min at 25 °C. Enzyme activity was calculated as µM TNB formed/min/mg protein (Extinction coefficient of TNB=13,600 M^−1^ cm^−1^).

*Glutathione peroxidase activity (EC 1.11.1.9):* Reaction mixture containing 1 mM EDTA in 50 mM potassium phosphate buffer (pH 7.0) was prepared. Cell lysates containing 40 µg protein samples were incubated with 340 µl reaction buffer, 60 µl 10 mM GSH, 60 µl glutathione reductase (2.4 U/ml) and 60 µl 1.5 mM NADPH at 37 °C for 3 min. Thereafter, reaction was initiated by adding 60 µl of 2 mM H_2_O_2_ and absorbance measured at 340 nm for 5 min at 60 s interval (Extinction coefficient for NADP^+^=6.22 mM^−1^ cm^−1^).

*NQO1 activity (EC 1.6.5.2):* NQO1 activity was assayed using a two-electron acceptor dichloroindophenol (DCIP). Reaction mixture containing 50 mM Tris–Cl (pH 7.5), 0.08% Triton X-100, 0.25 mM NADPH and 80 µM 2,6-dichloroindophenol (DCIP) was prepared. To an assay cuvette containing 40 µg/ml protein sample, the reaction started by the addition of 0.695 ml of reaction mixture. The two-electron reduction of DCIP was monitored kinetically at 600 nm at 25 °C for 3 min. Activity was calculated as nM DCIP reduced/min/mg protein (Extinction coefficient of reduced DCIP=21 mM^−1^ cm^−1^).

*Glutathione-sulfotransferase activity:* The activity was estimated using CDNB (1-chloro-2,4-dinitrobenzene), a synthetic GST substrate. The assay measures conjugation of CDNB with reduced glutathione. To 380 µl of 105 mM potassium phosphate buffer (pH 6.5), 4 µl 100 mM GSH was added followed by addition of cell lysate containing 40 µg protein sample. 4 µl of 100 mM CDNB (prepared in methanol) was added and change in absorbance recorded at 340 nm for 5 min at 60 s interval (Extinction coefficient of GS-DNB conjugate=9.6 mM^−1^ cm^−1^).

### 2.12 siRNA transfection

Silencer Select predesigned siRNA against rat Nfe2l2 (Nrf2) was obtained from Ambion. Transfection was performed in Opti-MEM Reduced Serum Medium (Gibco) 24 h after plating using Lipofectamine RNAiMAX Transfection Reagent in agreement with manufacturer's instructions. After 4 h incubation, the transfection medium was changed with serum-supplemented medium and the hepatocytes were further cultured for additional 20 h. Western blot analysis revealed an approximate 50–60% knockdown of Nrf2 post 24 h transfection with 50 nM siRNA concentration compared to negative control siRNA. A concentration of 50 nM siRNA was selected for further experiments. Stimulations by tBHP and morin were done 48 h after plating.

### 2.13 TransAM Nrf2-ARE binding assay

The Nrf2-DNA binding activity was measured using ELISA-based assay (TransAM kits, Active Motif, Carlsbad, CA) following the manufacturer's instructions. However, in case of in-vivo study, the nuclear lysates were prepared by lysing the nuclei obtained from liver tissue as per the method described above.

### 2.14 Immunoprecipitation and immunoblotting to detect Nrf2 ubiquitination

Non-denaturing immunoprecipitation was performed by bringing about the lysis of cells in 1 ml of radio immune precipitation assay buffer (RIPA) for 30 min at 4 °C, scraped followed with centrifugation at 16,000*g* for 20 min at 4 °C. The supernatant was rotated with anti-Nrf2 antibody overnight at 4 °C on rocker. Protein A/G PLUS (Santa Cruz Biotechnology) was added (30 μl), and samples were incubated for 2 h at 4 °C. Immunoprecipitates thereafter were pelleted, washed three times with 1 ml of radioimmune precipitation assay (RIPA) buffer, boiled in Laemmli buffer and were analyzed by western blotting with anti-ubiquitin antibody [7].

### 2.15 Immunoblot analysis

Hepatocytes were pelleted, washed with ice-cold phosphate-buffered saline (PBS) and lysed in NP-40 lysis buffer to obtain total cellular protein. The supernatant was collected by centrifuging at 17,000*g* for 15 min and stored in aliquots at −80 °C. Protein concentration was estimated using Bicinchoninic acid (BCA) method. 40–50 µg protein samples were resolved by 10% SDS-PAGE and thereafter transferred onto polyvinylidene fluoride (PVDF) membrane (Hybond-P, Amersham Biosciences, Piscataway, NJ). Immunoblot was visualized using Immobilon western chemiluminescent horseradish peroxidase substrate kit (Millipore Corporation, Billerica, MA, USA). Chemiluminescence signals were captured using Versa Doc image analyzer (Bio-Rad, Hercules, CA, USA) and protein expression levels were analyzed using ImajeJ 1.44p software (National Institute of Health, Bethesda, MD, USA).

### 2.16 Docking study

To get the insight into the binding mode of Morin with PP2C domain of PHLPP2, we performed docking of Morin to PP2C domain. The structure of Morin was built with sketch module of Sybyl7.1 (Sybyl, Version 7.1, Tripos, Inc., St. Louis, MO, 2005). The structure of Morin was geometrically optimized within Sybyl7.1 using MMFF94 force field and MMFF94 charges with 1000 iterations. AutoDock4.2 [17] was used to dock Morin into the binding site of homology model of PP2C domain of PHLPP2 protein that has been published by Sierecki et al. [18]. The molecular docking study of Morin with Fyn kinase was also performed using Autodock4.2 software. The crystal structure of Fyn kinase was obtained from RCSB protein data bank (Accession code: 2DQ7) [19]. All the structure visualization was performed using Chimera [20].

### 2.17 Morin-conjugation to sepharose beads and pull-down assay

*Preparation and coupling of beads with morin:* Approximately, 300 mg lyophilized CNBr-activated Sepharose 4B powder was weighed and suspended in 10 ml of 1 mM HCl. The swollen beads were thereafter washed for 15 min with 1 mM HCl (by giving 10 washes of 10 ml each) and finally with coupling buffer (0.1 M NaHCO_3_ pH 8.3, 0.5 M NaCl). The medium was equally divided into two, one for blank and the other for coupling morin. 2 mg morin was weighed and dissolved in 100 µL DMSO, thereafter coupling buffer was added to make up the volume to 1.5 ml. The solution of morin was added to the beads medium. To the medium labeled as blank, 1.5 ml of coupling buffer with 100 µL DMSO was added. The suspension was rocked overnight at 4 °C. Washing and blocking steps were performed as directed by the manufacturer. The entire process was followed with blank beads medium as well.

*Pull-down reaction:* The beads were washed twice with reaction buffer (50 mM Tris pH 7.5, 5 mM EDTA, 150 mM NaCl, 1 mM DTT, 0.01% NP-40, 2 µg/ml BSA, 0.02 mM PMSF and protease inhibitor cocktail). Approximately, 100 µg of cell lysate was incubated with 50 µl packed volume of both morin-conjugated as well as blank beads separately and suspended in reaction buffer. The tubes were rotated end-over-end overnight at 4 °C. The proteins pulled down along with the beads were washed thrice with reaction buffer, boiled in 2× SDS loading dye and applied to 10% SDS-PAGE along with a separate well for 50 µg cell lysate. The proteins were transferred onto PVDF membrane, which was later probed with antibodies against PHLPP2 and Fyn kinase.

### 2.18 Statistical analysis

All computational calculations of quantitative data were performed using Microsoft Excel program. Each experiment was repeated at least three times. The quantitative variables represented in histograms are expressed as mean±SEM. Statistical comparisons between means of different groups were conducted by one-way Analysis of Variance (ANOVA) followed by Tukey's post hoc test using SPSS 14.0 statistical package (SPSS Inc., Chicago, IL, USA). Differences were considered statistically significant when *P*<0.05.

## 3 Results

### 3.1 Morin directly protects hepatocytes against oxidative stress induced cell death

**Fig. 1:**
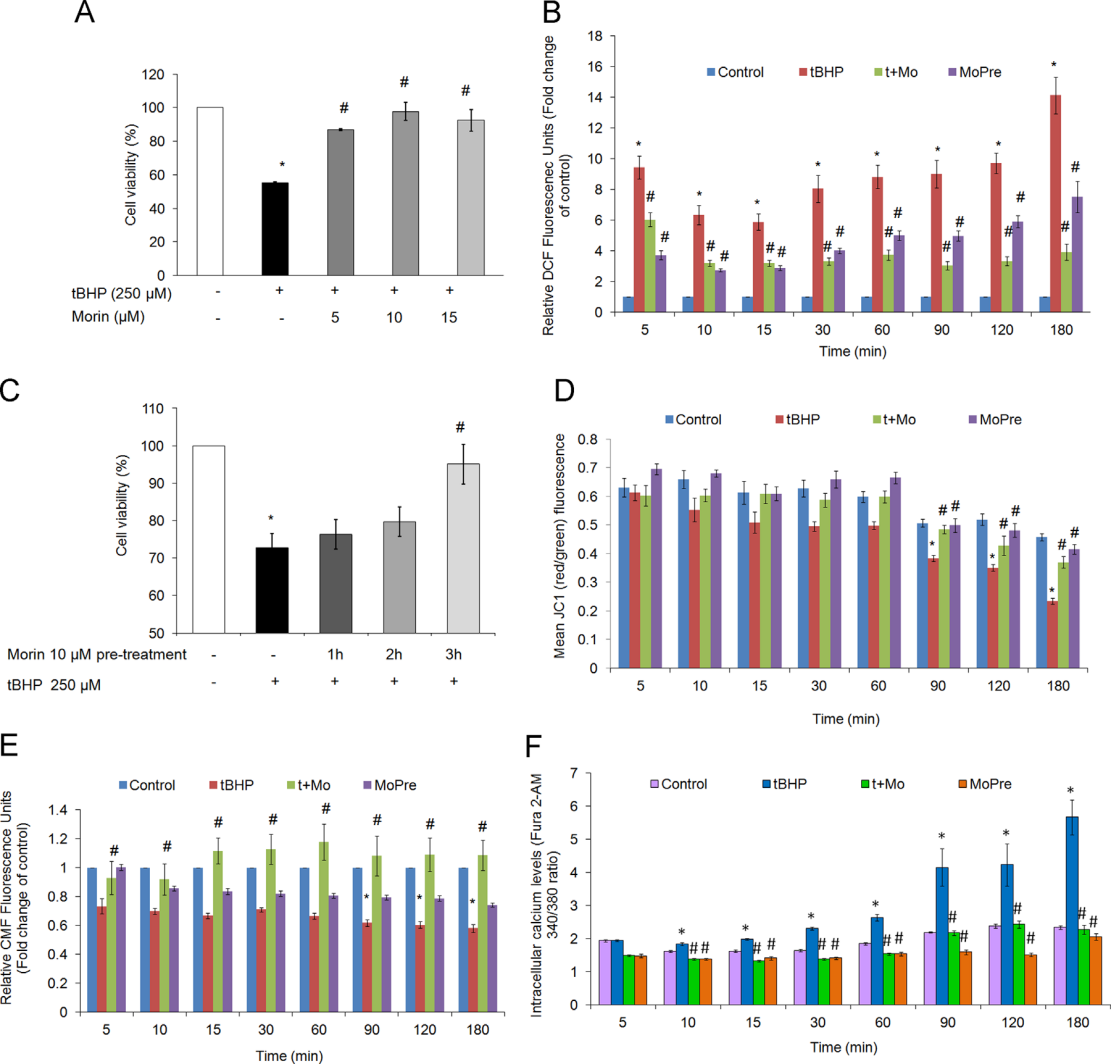
Morin prevents tBHP-induced death and oxidative stress in primary hepatocytes. Cell viability was determined using MTT assay in primary rat hepatocytes (1×10^4^ cells/well) treated with (A) 250 µM tBHP together with non-cytotoxic doses (5, 10 or 15 µM) of morin for 180 min, (B) time-dependent (1 h, 2 h and 3 h) exposure to 10 µM morin followed by 90 min treatment with 250 µM tBHP. The data are represented as mean±SEM of three independent experiments. *⁎P*<0.05 vs. control; #*P*<0.05 vs. tBHP treatment. Effect of morin in preventing (C) intracellular ROS generation and (D) mitochondrial membrane permeabilization was assessed by incubating hepatocytes with 10 µM DCFH-DA or 5 µM JC-1 respectively for 30 min at 37 °C. Fluorescence was determined at the mentioned time-points upto 3 h. (E and F) Morin maintains GSH pool and intracellular calcium levels of tBHP-treated primary hepatocytes. Hepatocytes were treated with 5 µM CMFDA (E) or 5 µM Fura 2-AM (F) for 30 min at 37 °C. The cells were rinsed and treated with 250 µM tBHP (tBHP) for 90 min either in combination with 10 µM morin (t+Mo) or following prior 3 h incubation with 10 µM morin (MoPre). Fluorescence was determined at the mentioned time-points upto 3 h. The data are represented as mean±SEM of three independent experiments. *⁎P*<0.05 vs. control*; #P*<0.05 vs. tBHP treatment.

To explore the key protective mechanism behind its role as an antioxidant, we investigated whether morin could protect isolated hepatocytes from toxicity generated due to oxidative overload. For this we employed tBHP (*tert-*butyl hydroperoxide), a pro-oxidant, wherein GSH depletion is believed to be a prime causative factor in the cytotoxicity ensued by oxidative stress [21]. 250 µM concentration of tBHP at 90 min exposure was selected for studies involving cytoprotection by morin since this concentration was observed to establish significant cell death due to oxidative stress [7] which is indicative of collapse in cellular defensive mechanisms. Three different non-cytotoxic concentrations of morin, 5 µM, 10 µM and 15 µM, were co-administered with 250 µM tBHP. Co-treatment of hepatocytes with 10 µM morin exhibited significant improvement in the cell survival rate reaching 97.7% (*P*<0.05; Fig. 1A). For pre-treatment studies with morin, a pre-incubation period of 3 h was found to be effective in exerting protective effects upon subsequent 90 min exposure of 250 µM tBHP (Fig. 1B) which increased the viability of hepatocytes to 93.5%. The improved cell survival capacity was observed to be a result of suppressed intracellular ROS levels as estimated by DCF fluorescence (Fig. 1C). Apart from preventing collapse of mitochondrial membrane potential (Fig. 1D), treatment with morin also precluded alterations inGSH and intracellular calcium levels (Fig. 1E and F).

### 3.2 Morin prevents tBHP-evoked modulation in levels of pro-apoptotic and Erk-JNK proteins

**Fig. 2:**
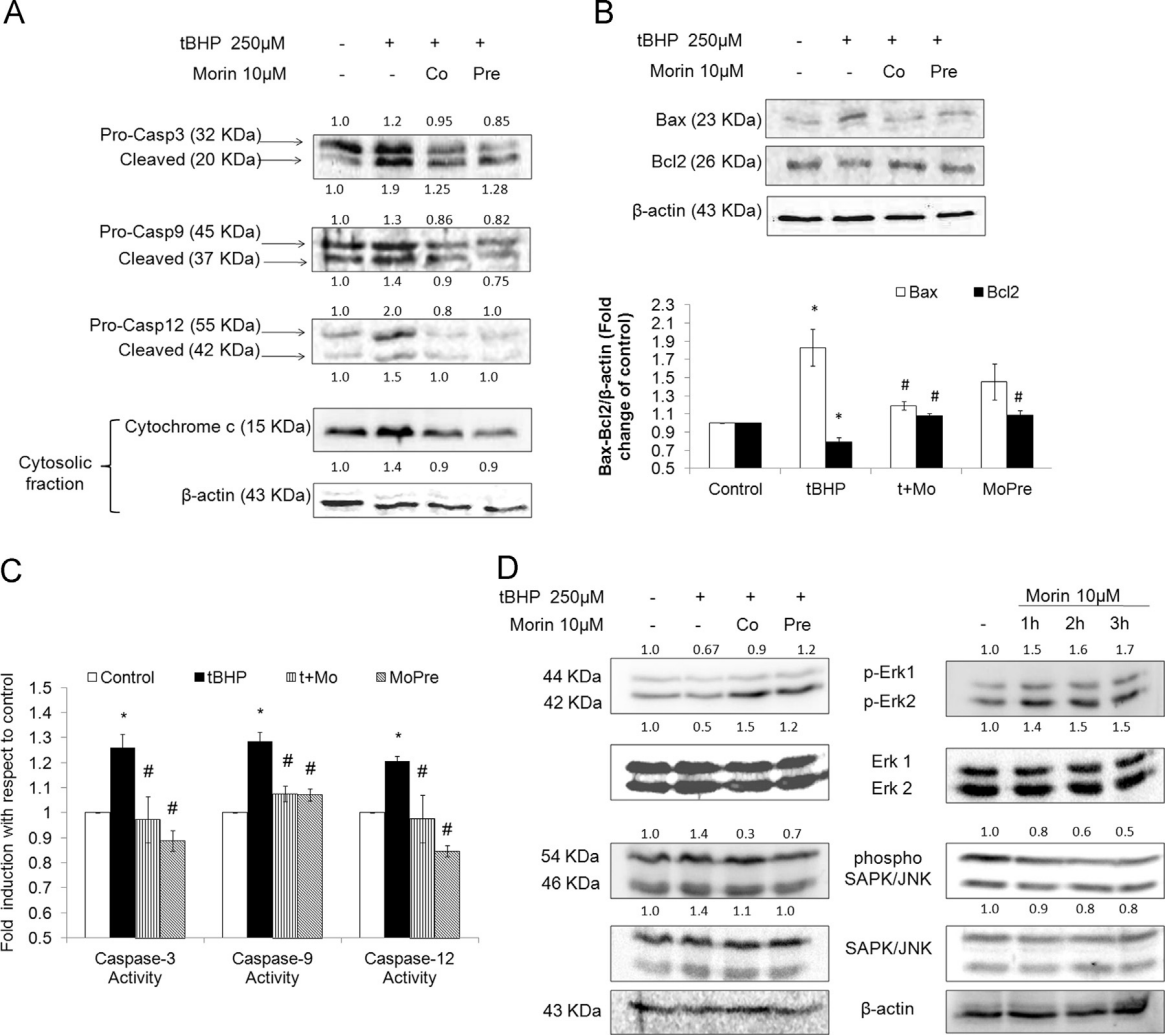
Morin modulates tBHP-mediated oxidative stress and the consequent alteration in protein levels of cell death pathways. Primary rat hepatocytes were treated with 250 µM tBHP (tBHP) for 90 min and either in combination with 10 µM morin (t+Mo) or following prior 3 h incubation with 10 µM morin (MoPre). (A) Western blot images depict levels of caspase-3, caspase-9, caspase-12, Bax and Bcl2 in whole cell extracts and cytochrome c in cytosolic extracts. Results were normalized to β-actin. Values indicate fold change in band density relative to respective control values. (B) Activity of caspase-3, caspase-9 and caspase-12 estimated in cytosolic extracts using colorimetry or fluorimetry based assays. (C) Representative immunoblots showing effect of morin on activation of Erk1/2 and SAPK/JNK in primary rat hepatocytes. *β*-actin served as a loading control. The change in phosphorylation was quantitated relative to their non-phosphorylated forms, following which; fold change in band density was obtained relative to respective control values. The data are represented as means±SEM of three independent experiments. *⁎P*< 0.05 vs. control; *#P*<0.05 vs. tBHP/APAP treatment.

While loss of mitochondrial membrane potential results in sequential activation of caspase-9 and caspase-3, activation of caspase-12 is a key hallmark of endoplasmic reticulum stress accompanied by alterations in calcium homeostasis. The reduction in oxidative burden correlated well with suppression of tBHP-evoked caspases activation (Fig. 2A and B). While morin co-treatment appreciably prevented Bax accumulation, both morin co- as well as pre-treatments enhanced cellular Bcl2 levels apart from lowering tBHP-mediated increase in cytosolic cytochrome c levels (Fig. 2A). Erk1/2 can regulate cell survival and apoptotic mechanisms by influencing the activity of anti- and pro-apoptotic transcription factors [22]. Moreover, its phosphorylation levels are found to be negatively influenced by pro-oxidant exposures [23], [24]. The decrease in Erk1/2 phosphorylation and increased phosphorylation of SAPK/JNK in response to tBHP stimulus was prevented by morin supplementation (Fig. 2C). The potential of morin to influence molecular signaling events can be gauged from the observation that treating hepatocytes with morin alone also increased phosphorylation of Erk1/2 while reducing that of SAPK/JNK in a time-dependent manner (Fig. 2C). Studies involving phytochemicals like sulforaphane [25], quercetin [26], genistein [27] have shown that activation of MAP kinases like Erk, JNK or PI3K/Akt pathway may be involved in Nrf2 transactivation which subsequently fortifies cellular antioxidant defenses. The results, in aggregate, demonstrate that morin's capacity to limit oxidative stress plays a key role in promoting cytoprotection against oxidant challenge apart from its potential to limit ER stress ensued damage via Erk-Nrf2 pathway [28].

### 3.3 Morin induced cytoprotection against oxidative stress is Nrf2-regulated

**Fig. 3:**
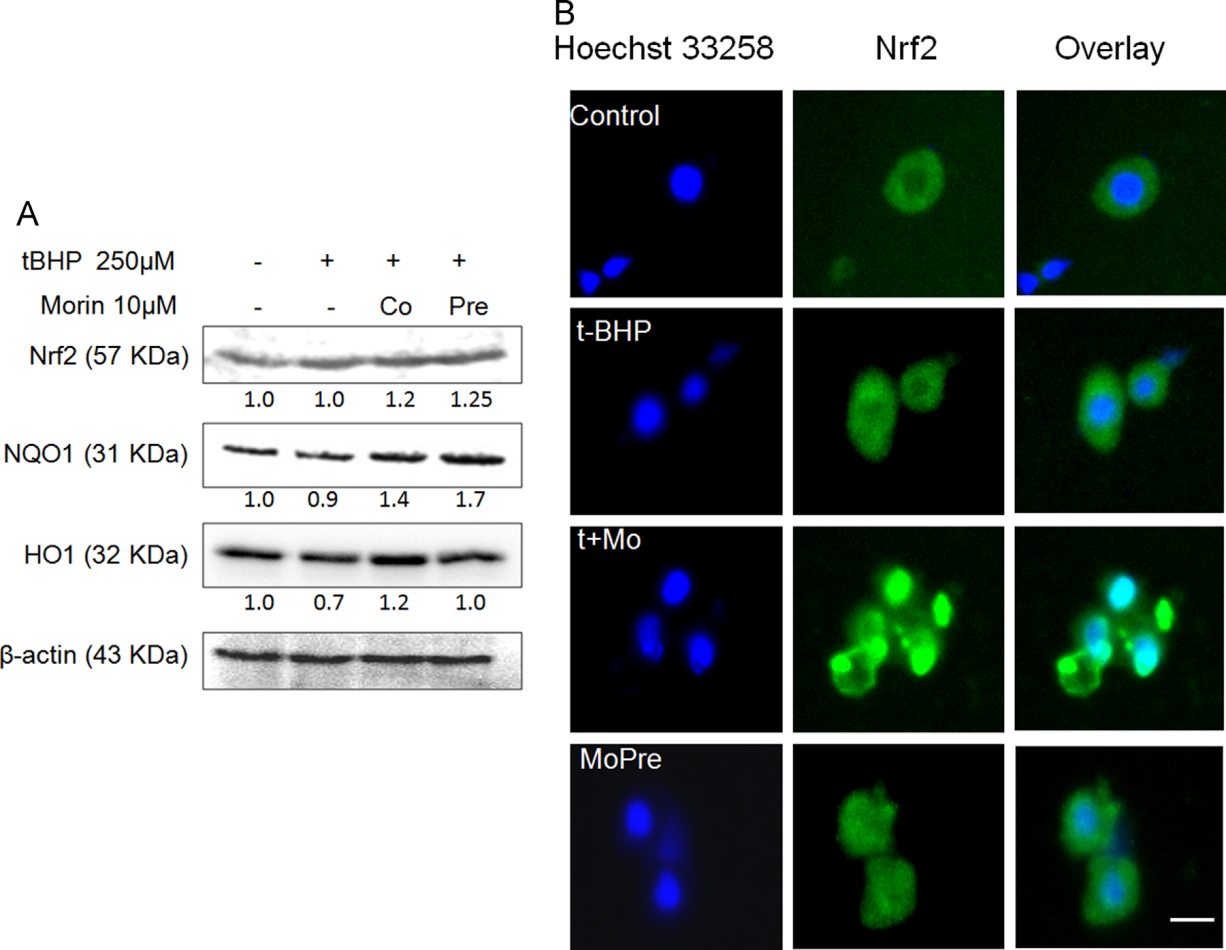
Morin counteracts tBHP-evoked oxidative damage by enhancing nuclear accumulation of Nrf2. (A and B) Primary rat hepatocytes were treated with 250 µM tBHP (tBHP) for 90 min and either in combination with 10 µM morin (t+Mo) or following prior 3 h incubation with 10 µM morin (MoPre). (A) Effect of morin on protein expression of Nrf2 and its downstream targets. (B) Immunofluorescent staining of primary hepatocytes depicting Nrf2-compartmentalization in hepatocytes. Scale bar, 20 µm.

In our earlier report [7], we have demonstrated the contribution of suppressed Nrf2 responses to deterioration of cellular defense system, ultimately resorting to death of oxidant-exposed hepatocytes. We reckoned that the cytoprotective efficacy of morin against oxidative damage might be a manifestation of its capacity to potentiate Nrf2-regulated survival pathway. Treatment of tBHP-stressed hepatocytes with morin displayed a consistent increase in the protein level of target enzymes of Nrf2 that is, NQO1 and HO1 (Fig. 3A). Microscopic evaluation revealed notable retention of Nrf2 within the nucleus upon co-treatment with morin as indicated by enhanced fluorescence under green channel corresponding to the nuclear region depicted by Hoechst staining (Fig. 3B, t+Mo). In case of pre-treatment, the density of Nrf2 within the nucleus exhibited considerable improvement over tBHP-treated hepatocytes but was less pronounced as compared to co-treated cells.

### 3.4 Morin promotes nuclear retention of Nrf2 by promoting its stability

**Fig. 4:**
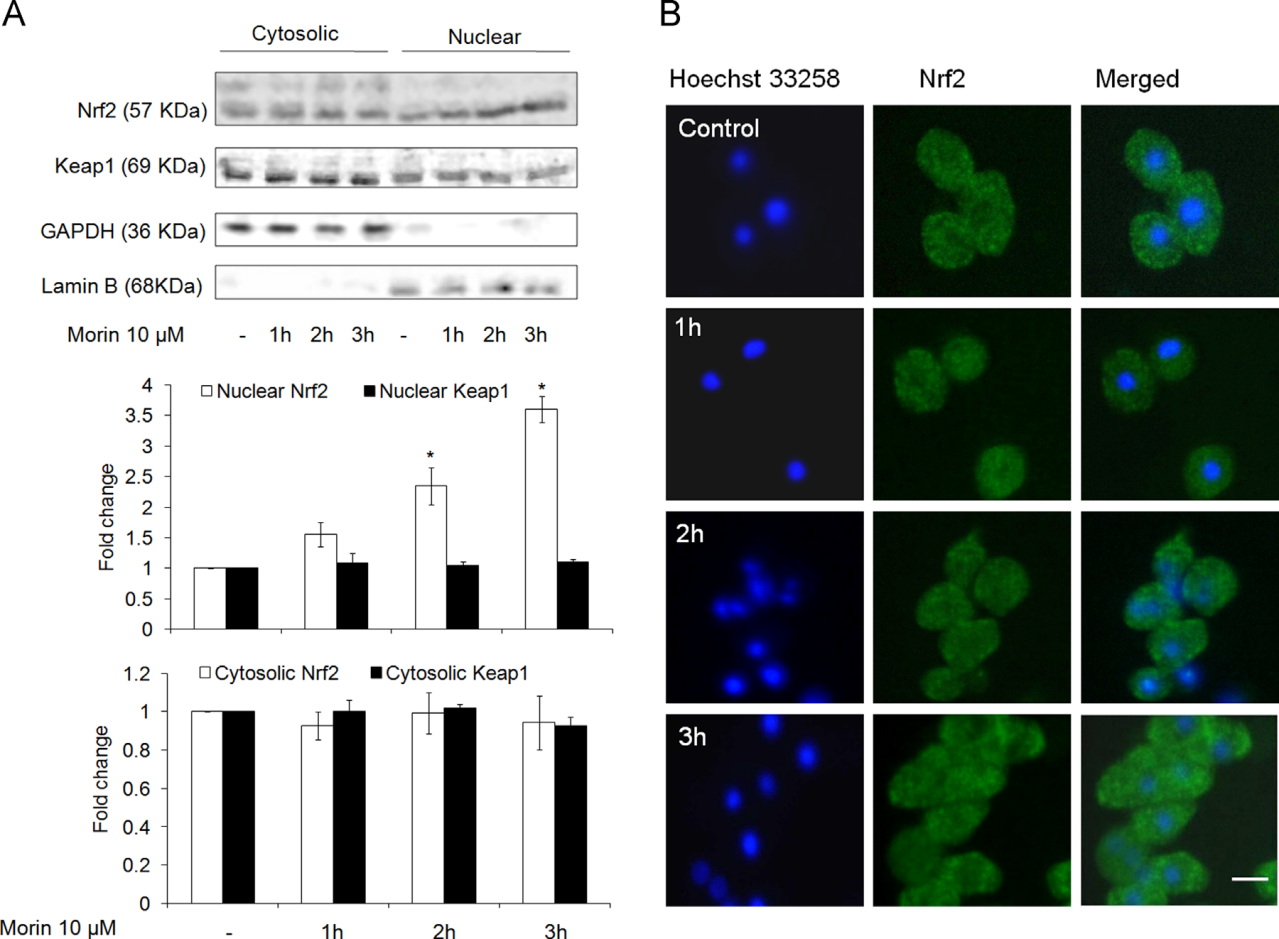
Morin promotes nuclear retention of Nrf2. (A) Western blot analysis and (B) Immunofluorescent staining of primary hepatocytes to depict Nrf2-compartmentalization in hepatocytes treated with 10 µM morin alone for 1 h, 2 h and 3 h. Scale bar, 20 µm. The images are representative of three independent experiments. The data are represented as means±SEM of three independent experiments. *⁎P*<0.05 vs. control;

To determine whether morin is individually capable of inducing nuclear translocation of Nrf2, we studied the distribution of Nrf2 in nuclear and cytoplasmic fractions of unstressed hepatocytes treated with 10 µM morin alone for 1 h, 2 h and 3 h. Western blot analysis revealed time dependent increase in nuclear Nrf2 density with approximately 3.6 fold increase at 3 h exposure (Fig. 4A). Distribution of Nrf2 in the cytoplasmic fraction, however, showed no change with time. Immuno fluorescent detection also supported the western blot data (Fig. 4B). Further, morin did not alter the levels of Keap1 in both cytosolic and nuclear fractions (Fig. 4A). This signifies that morin may have promoted nuclear accumulation of Nrf2 via a pathway distinct from Keap1-regulated Nrf2 turnover.

**Fig. 5:**
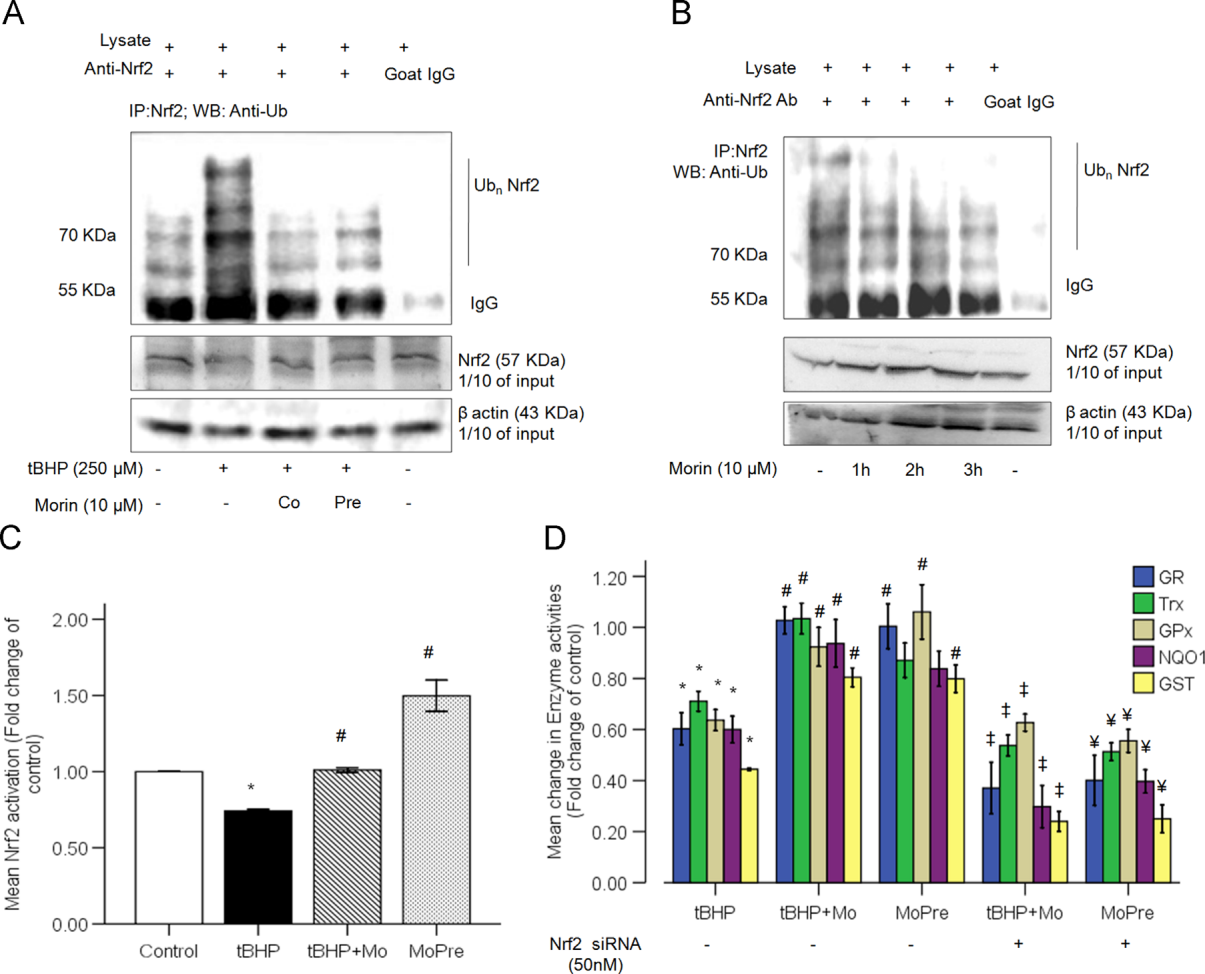
Morin counteracts oxidative damage by promoting Nrf2 stability and activation, thereby strengthening cellular antioxidant defenses. (A) Nrf2 ubiquitination status was estimated in total cell lysates obtained from tBHP and/or morin treated primary rat hepatocytes as indicated. Nrf2 was immunoprecipitated with anti-Nrf2 antibody followed by immunoblotting with anti-ubiquitin antibody. A negative control where anti-Nrf2 antibody was replaced with normal goat serum was also included. (B) Nrf2 ubiquitination status in hepatocytes treated with 10 µM morin alone for 1 h, 2 h and 3 h. (C) ELISA-based Nrf2 binding activity in nuclear extracts of treated primary rat hepatocytes. (D) Enzyme activities were determined in cell lysates obtained from untransfected hepatocytes treated with tBHP and/or morin as well as hepatocytes transfected with 50 nM Nrf2 siRNA and thereafter exposed to tBHP and/or morin 24 h post-transfection. GR: glutathione reductase; Trx: thioredoxinreductase; GPx: glutathione peroxidase; GST: glutathione sulfotransferase. The data are represented as means±SEM of three independent experiments. *⁎P*<0.05 vs. control; *#P*<0.05 vs. tBHP treatment; *‡P*<0.05 vs. respective t+Mo treatments of untransfected cells; *¥P*<0.05 vs. respective MoPre treatments of untransfected cells.

Apart from nuclear import, there are other pathways that distinctly operate to assist nuclear retention of Nrf2, such as the one responsible for determining Nrf2 stability. Previous studies have indicated that enhanced Nrf2 stability transpires increased Nrf2-activation [29]. As was anticipated, tBHP stress induced considerable ubiquitination of Nrf2 (Fig. 5A) which was prevented by morin. Furthermore, treatment of hepatocytes with morin alone was also observed to reduce levels of endogenously ubiquitinated Nrf2 (Fig. 5B). Treatment with tBHP significantly reduced the ARE-binding efficacy of Nrf2 by 25% in comparison to control. While co-treatment with morin effectively maintained the Nrf2 transcriptional activity comparable to control, pre-treating the hepatocytes with morin prior to tBHP-evoked oxidative stress led to a significant 2 fold increase in nuclear Nrf2-binding activity as compared to tBHP (Fig. 5C). Further, morin significantly improved the tBHP-induced decline in activities of redox and phase-II enzymes in wild-type hepatocytes (Fig. 5D, tBHP+Mo and MoPre). The activities of antioxidant enzymes were further studied in Nrf2-silenced hepatocytes treated with morin and tBHP (Fig. 5D). The results revealed significant decrease in the activities of all the enzymes during in Nrf2-silenced hepatocytes due to tBHP exposure irrespective of morin treatment (co- or pre-). This suggests that the reinforcement of the antioxidant defenses of cell due to morin treatment is not a result of its antioxidant potential alone but due to potentiation of Nrf2-regulated transcriptional induction. We may thus assume that Nrf2-mediated up-regulation of antioxidant and detoxification enzymes observed due to morin can be explained, at least in part, by enhancement of Nrf2 stability leading to its increased nuclear retention and ARE-Nrf2 binding affinity.

### 3.5 Suppression of PHLPP2 activity accounts for cytoprotective efficacy of morin against oxidant stress

**Fig. 6:**
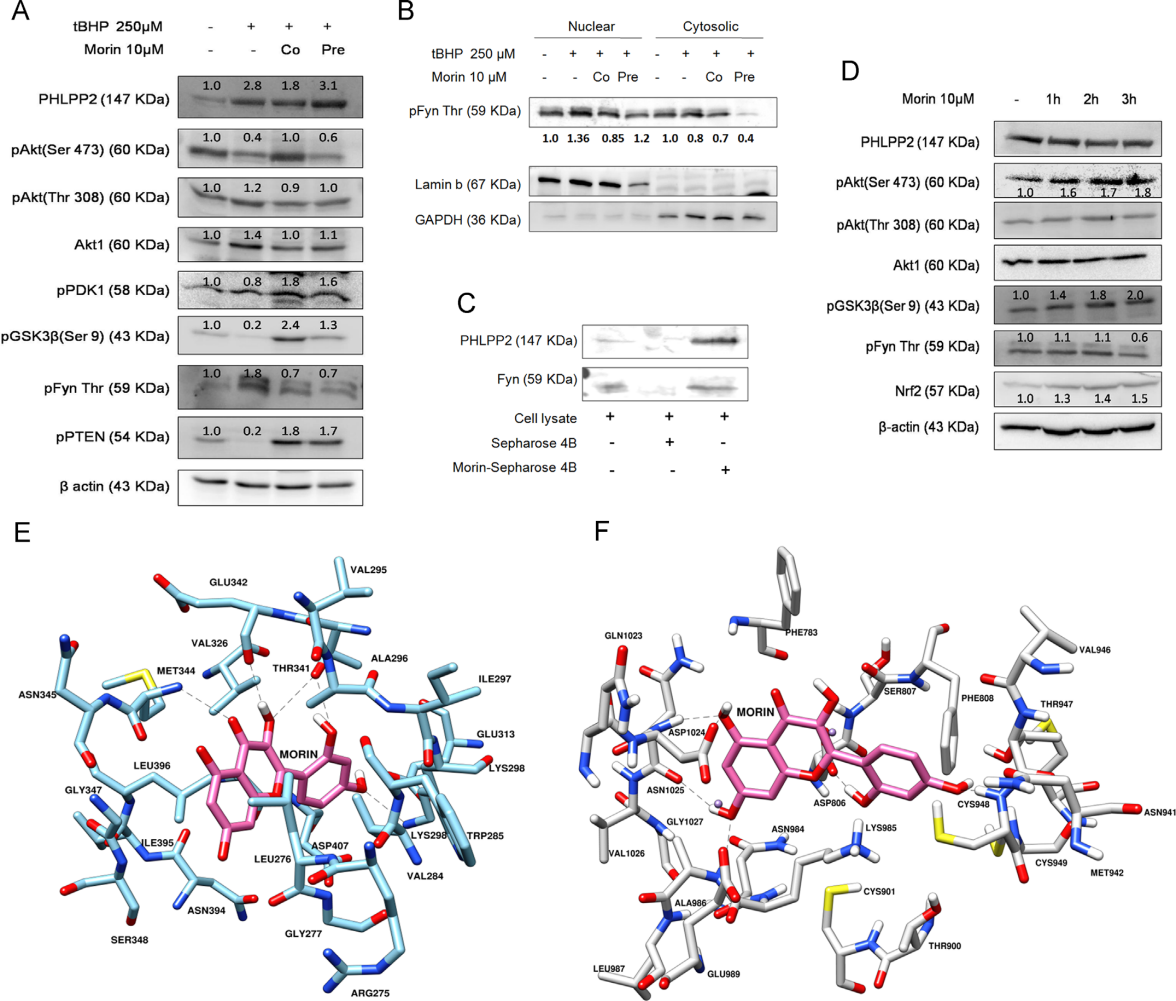
Morin shows structural affinity towards PHLPP2 and intervenes in PHLPP2-mediated dephosphorylation of downstream effectors. The potential of morin to modulate key proteins of PHLPP2-regulated pathway was studied and confirmed in tBHP treated hepatocytes. Primary rat hepatocytes were treated with 250 µM tBHP (tBHP) for 90 min and either in combination with 10 µM morin (t+Mo) or following prior 3 h incubation with 10 µM morin (MoPre) and key proteins of PHLPP2-regulated pathway were assessed using western blot analysis. (A) Estimation of protein levels was done in total tissue lysates of tBHP-treated or morin supplemented hepatocytes. *β*-actin served as a loading control. (B) Relative levels of phospho-Fyn kinase in the nuclear and cytosolic fractions obtained from hepatocytes treated with tBHP and/or morin. Lamin-b served as loading control for nuclear samples while GAPDH served as a loading control for cytosolic samples. (C) Pull-down assay demonstrating PHLPP2 and Fyn kinase binding capacity of morin. CNBr-activated Sepharose beads conjugated with morin were rocked with tissue lysates. Western blots were probed with anti-PHLPP2 and anti-Fyn kinase antibody. Un-conjugated sepharose beads were also included in the experiment to detect non-specific binding of protein to beads. (D) Intervention of morin in PHLPP2-regulated pathway was confirmed in hepatocytes treated with 10 µM morin alone for 1 h, 2 h and 3 h followed by protein isolation and western blot analysis where β-actin has been used as endogenous control. The data are representative of three independent experiments. (E) Interaction of Morin with Fyn kinase. The protein residues are shown in Cyan color and Morin is shown in Pink, dash lines represents hydrogen bonds. (F) Interaction of Morin with PP2C domain of PHLPP2. The protein residues are shown in Grey color and Morin is shown in Pink. Dash lines represent hydrogen bonds. Magnesium ions are shown as purple color spheres. (For interpretation of the references to color in this figure legend, the reader is referred to the web version of this article.)

We have previously proven and discussed the involvement of Fyn kinase in determining Nrf2 stability via PHLPP2-Akt (Ser473)-GSK3β pathway [7]. Since, morin treatment was observed to attenuate Nrf2 ubiquitination, we reckoned that the protection accorded by morin, by way of potentiating Nrf2 signaling, might involve modulation of PHLPP2-regulated Fyn kinase activation. In agreement to our hypothesis, morin prevented tBHP-induced phosphorylation of Fyn kinase by limiting PHLPP2-mediated deactivation of Akt at Ser473 residue (Fig. 6A). This in turn inactivated GSK3β, which promotes Fyn kinase phosphorylation when active. Nuclear levels of phosphorylated Fyn kinase were also reduced by morin supplementation (Fig. 6B). Pull-down assay evidenced the structural interaction of morin with both Fyn kinase and PHLPP2 which are negative upstream modulators of Nrf2-activation (Fig. 6C).

Treatment of hepatocytes with 10 µM morin alone for increasing time periods (1 h, 2 h and 3 h) did not have any effect on PHLPP2 expression as revealed by immunoblot experiments (Fig. 6D). However, significant increase upto 1.8 fold (*P*<0.05) in phosphorylation of Akt at Ser473 residue could be detected at 3 h. Further, phosphorylation of GSK3β was also enhanced in a time-dependent manner reaching 2 fold at 3 h in comparison to control (Fig. 6D). Morin treatment for 3 h also significantly suppressed phosphorylation of Fyn kinase by 42% (*P*<0.05). These changes were accompanied by time-dependent increase in cellular Nrf2 levels. The experimental evidence from western blot analysis reveals that morin could exert notable intervention in Nrf2 signaling pathway by preventing Fyn-kinase phosphorylation and hence reduced Nrf2 degradation.

Morin was docked to ATP-binding site of Fyn kinase domain in complex with an inhibitor staurosporine (Fig. 6E). The binding mode of morin in docked conformation with Fyn kinase was found similar to that observed in the crystal structure. Morin was observed to be involved in several hydrogen bonds with Met344, Thr341, Glu342 and Lys298 residues in the ATP-binding site of Fyn kinase. It has been reported earlier that these interactions play important role in ligand-binding to Fyn kinase [30]. The docking experiment corroborates the previous finding where morin was shown to inhibit Fyn kinase activity [31]. The findings would collectively suggest that Fyn kinase can serve as a potential target of morin in potentiation of Nrf2 pathway. However, we observed that morin limited PHLPP2-mediated deactivation of Akt (Fig. 6A) which lies upstream of Fyn kinase axis. Furthermore, treatment of hepatocytes with morin alone induced noticeable increase in phosphorylation of Akt(Ser473) and subsequent targets, without any apparent alteration in PHLPP2 levels (Fig. 6D). This led us to posit that morin could have exerted positive influence on Akt activation, and hence Nrf2 signaling, by affecting phosphatase activity of PHLPP2, as indicated by pull-down experiment (Fig. 6C). In an attempt to examine such a possibility, morin was docked into the binding site of the homology model of PP2C domain of PHLPP2 as identified by Sierecki et al. [18]. Morin docked well in this binding site with binding energy of −6.27 kcal/mol. As shown in Fig. 6E, morin interacts with Asp1024 and Glu989 by hydrogen bonds in docked complex. Besides these, it also interacts with Ala986, Asn1025 and Cys948 through hydrogen bonds (Fig. 6F). These interactions have previously been reported important for inhibition of activity of PP2C domain [18]. The cumulative findings thus indicate that the inhibition of PHLPP2 through structural interaction of morin might be involved in averting suppression of Nrf2 responses by oxidant exposure.

### 3.6 Morin prevents acetaminophen induced toxic effects in vitro

**Fig. 7:**
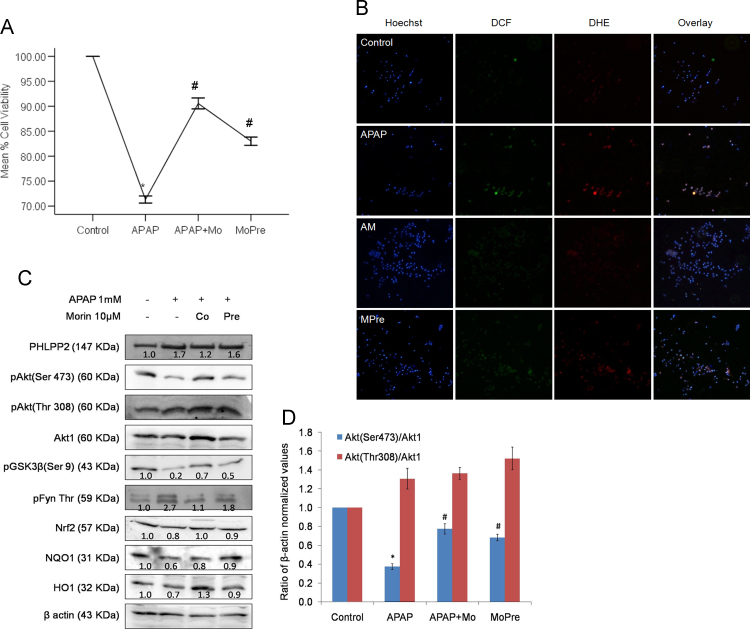
Morin prevents acetaminophen-mediated cytotoxic effects on primary hepatocytes through intervention in PHLPP2-regulated down-modulation. Acetaminophen-induced toxicity was estimated in primary rat hepatocytes treated with 1 mM APAP for 90 min. Morin (10 µM) was added either together with (AM) or 3 h prior to APAP treatment (MPre). (A) The change in cell viability was measured using standard MTT assay. The data are represented as means±SEM of three independent experiments. *⁎P*<0.05 vs. control; *#P*<0.05 vs. tBHP/APAP treatment. (B) ROS levels were assessed using fluorescence microscopic estimation of DCF/DHE staining. (C) Key proteins of PHLPP2-regulated pathway were assessed using western blot analysis. Estimation of protein levels was done in total tissue lysates of APAP-treated or morin supplemented hepatocytes. *β*-actin served as a loading control.

To further validate the findings, the protective action of morin involving Nrf2 potentiation was dissected in a separate model of oxidative stress-induced hepatocellular toxicity. Acetaminophen has previously been reported to cause oxidative damage and subsequent cell death in primary rat hepatocytes [32]. Isolated primary rat hepatocytes were exposed to acetaminophen wherein GSH depletion is proposed to be a critical event in propagating cellular damage. The concentration of morin (10 µM) found potent against tBHP was assessed for its efficacy in managing acetaminophen (APAP)-induced cell death in-vitro. Apart from rescuing hepatocytes from cell death caused by APAP challenge (Fig. 7A), morin also reduced cellular ROS levels (Fig. 7B). Treatment with 1 mM APAP for a period of 90 min led to a significant (*P*<0.05) 30% reduction in viability of hepatocytes which was prevented by both morin co- and pre-treatment. While simultaneous exposure to morin improved the viability by 28.2% (*P*<0.05), pre-treatment enhanced cell-survival by 18.4% over that of APAP-treated values (Fig. 7A). The enhancement in the intensities of green (DCF) and red (DHE) channels detected in APAP-treated cells was significantly suppressed in morin co-treated hepatocytes (Fig. 7B). Morin pre-treatment also improved cellular ROS status but the effect was less pronounced as compared to co-treated hepatocytes. The results demonstrate that morin's capacity to contain oxidative stress plays a key role in promoting cytoprotection against APAP-challenge.

The role of morin as small molecule modulator of Nrf2 signaling pathway was assessed by investigating protein expression of key targets of PHLPP2-regulated pathway. Western blot analysis (Fig. 7C) revealed 2.7 fold increase in Fyn kinase phosphorylation together with near 80% decline in GSK3β phosphorylation in APAP-treated hepatocytes in comparison to control. This was associated with nearly 61% decrease in pAkt (Ser473) and 1.7 fold induction of PHLPP2 protein. APAP treatment also down-modulated the protein levels of Nrf2 along with subsequent inhibitory effects on protein expression of its downstream targets NQO1 and HO1 (Fig. 7C). Thus, the experiments employing APAP exhibited similar pattern of activation/deactivation of key proteins involved in PHLPP2 pathway as observed earlier with respect to tBHP. Supplementation with morin imparted significant restorative effects on the altered expression of the above proteins. The effect of morin co-treatment was more pronounced as can be seen from the 30% (*P*<0.05) reduction in PHLPP2 protein as compared to APAP-treated cells. Further, the expression of pAKT (Ser473) in APAP-treated cells was significantly enhanced in morin co-treated hepatocytes together with a profound 71.4% increase in phosphorylation of GSK3β as compared to APAP treatment. The levels of pFyn and Nrf2 were also restored to near control values. Thus, morin significantly attenuated the regulation imposed by PHLPP2 on Fyn kinase activation during APAP-induced oxidative toxicity imposed on primary rat hepatocytes.

## 4 Discussion

Oxidative stress has been established as a key factor in hepatotoxicity related disorders [33]. Liver, owing to its inherent function, is under constant risk of xenobiotic stress which is often claimed to be the primary cause of liver failure. It has long been believed that supplementation of diet with healthy phytonutrients may help in the maintenance of liver health. Phytochemicals are no longer considered mere scavengers of free radicals, but targeted interventions of molecular pathways are increasingly being identified with their physiological effects [34]. The structural characteristics of flavonoids not only account for their radical scavenging activity [35] but also their propensity to modulate activity of key elements of cell signaling pathways. Hou and Kumamoto [36] have extensively discussed how different flavonoids can serve as chemopreventive agents by directly binding to and inhibiting multiple kinases of distinct molecular pathways.

**Fig. 8:**
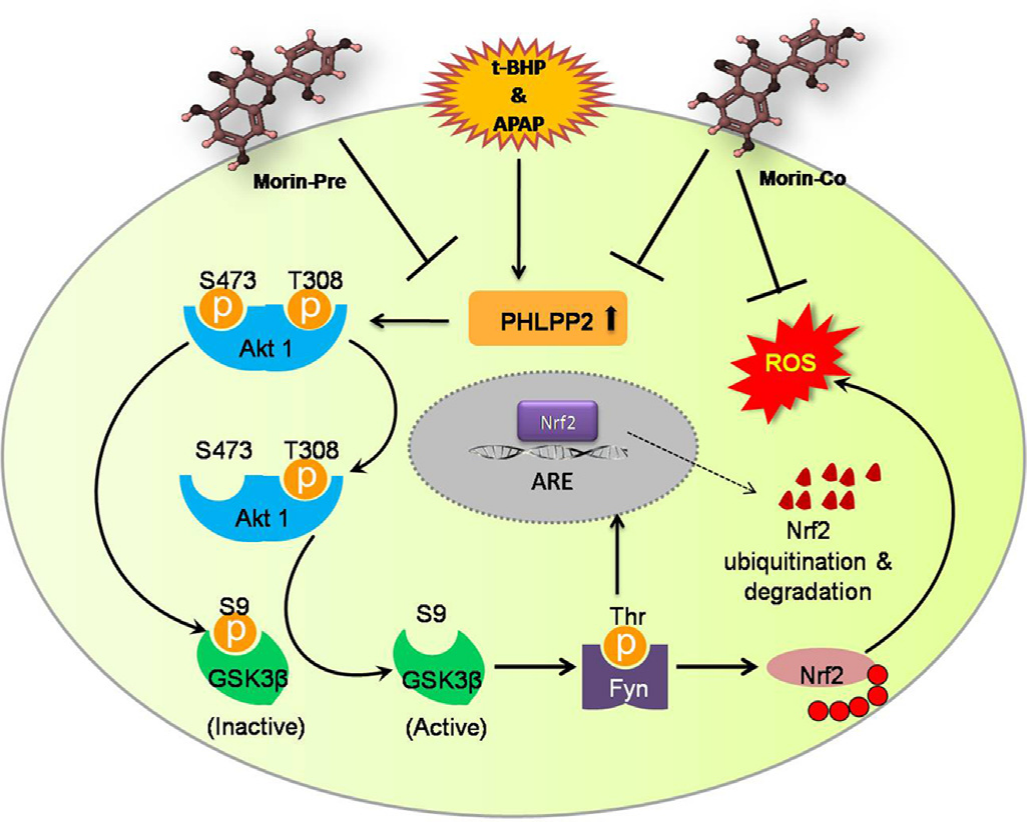
Scheme showing mechanism of Nrf2 modulation due to stress/morin treatment. Under stress conditions (tBHP/APAP), PHLPP2 induction leads to dephosphorylation of Akt1 at Ser473 residue which lifts the repression exerted by Akt on GSK3β. Activated form of GSK3β thereby promotes Fyn kinase activation which checks Nrf2 levels by mediating its degradation. This leads to ROS build-up and related toxicological implications. When morin is administered together with stress (Morin-Co), the protective effects exerted by the phytochemical entail both Nrf2 up-regulation as well as ROS quenching, since morin itself acts as an antioxidant. In case of addition of morin prior to stress application (Morin-Pre), substantial attenuation of oxidative stress is achieved due to down-modulation of PHLPP2-regulated Nrf2 suppression.

The present study provides fresh set of data, which further corroborates the notion that the impact of phytochemicals on physiology is in fact an outcome of direct influence on molecular signaling events. Morin appears to exert its cytoprotective activity by direct interplay with the signaling pathway regulating Nrf2 stability (Fig. 8). PHLPP2 expression is related to increased susceptibility to cell death. In our previous study [7], we have explored an association between the toxic implication of PHLPP2 induction and suppression of Nrf2 responses in the liver cells. Akt activation (its phosphorylation at Ser473 in particular) is known to inhibit GSK3β by phosphorylating Ser9 residue. This event prevents Fyn kinase activation, which, in its active form, is believed to mediate Nrf2-suppression via its ubiquitination [30]. In the present study, PHLPP2, a phosphatase that exclusively dephosphorylates Akt at its Ser473 residue, has been identified as a novel target of morin (Fig. 8). By binding to its active domain, morin subdues the phosphatase activity of PHLPP2. This lifts the repression imposed by PHLPP2 on Akt thereby stabilizing Nrf2.

Morin has been found to modulate varied protein targets that may translate into distinct response in different experimental settings. Strong inhibition of Organic Anion Transporter 1 (OAT1) by morin [37], for example, identifies its merit in lowering OAT1-mediated drug nephrotoxicity. Tyrosinase [38], GSK3β [39] and Fyn kinase [31] are other recent additions to the spectrum of cellular targets of morin. The capacity of morin to bind multiple protein targets may explain how morin exerts a wide range of physiological functions in our body. Interestingly, structural intervention of morin on PHLPP2 (present study) and that on Fyn kinase (Fig. 6E) [31] and GSK3β [39], all of which represent upstream modulators of Nrf2 signaling, may indicate the immense potential of morin to ensure activation of cell survival mechanism. Since each of the identified targets of morin is regulated by or regulates multiple other pathways, the selectivity of morin for a particular enzyme may be subject to the relative levels of each target. In other words, selectivity of a phytochemical for its probable targets may depend on context-dependent activation of specific signaling pathways as has been suggested earlier [36]. Given the strong antioxidant activity combined with its property to potentiate cellular survival mechanisms, morin may serve as a prospective candidate in designing a combination therapy along with drugs that pose significant chronic or acute toxic effects on liver. Administration of morin to rats in combination with non-steroidal anti-inflammatory drug, indomethacin, as a therapy for rheumatoid arthritis yielded better beneficial effects than either of the drug alone [40]. With the present study we may affirm that by way of conditioning deleterious responses, we would be in a position to extract more benefit out of drugs that may elicit oxidative injury to liver.

In summary, low redox signals fortify the cellular antioxidant defenses to prevent initiation of a consequent cell death cascade. However, if the oxidative burden rises to unmanageable levels, it may produce cytotoxic effects by destabilizing Nrf2 [7]. The study suggests that morin may adopt two independent modes to curtail redox imbalances and consequent damage to cellular moieties. In an event of co-administration with positive oxidative stress inducer, active participation of morin in free radical scavenging activity together with prevention of Nrf2 degradation could have led to subsidence of ROS burden. Experiments involving pretreatment with morin preclude the possibility of direct interaction with the oxidant or free radical species but given the potency of morin to influence cell signaling pathways, pre-exposure appears to prime Nrf2 for a rapid antioxidant defense build-up to counteract a subsequent challenge. Our study identifies a novel role of morin as a small molecule modulator of redox-sensitive signaling pathways apart from its antioxidant or free-radical scavenging activity. Pull-down and docking experiments reveal morin's potency to interact with PHLPP2 and influence PHLPP2-regulated site-specific activation of Akt thereby limiting Fyn kinase activation and its nuclear translocation. Hence, morin augments cell defense mechanisms through Nrf2 stabilization as suggested by silencing experiments wherein morin failed to mount antioxidant defenses against oxidative stress in Nrf2-knockdown hepatocytes. The mitigation of acetaminophen-induced hepatocytes toxicity by morin through intervention in PHLPP2-regulated pathway further corroborates its Nrf2-potentiating role. Apparently, the chemo-preventive mechanism followed by morin suggests that, beyond its antioxidant function, morin is involved in modulation of signaling pathways that promote Nrf2 stability which suggests that morin may be exploited to develop novel therapeutic strategy aimed at enhancing Nrf2 responses that could address toxicological changes associated with xenobiotic stress.

## Conflict of interest

The authors declare no conflict of interest.
